# Fine-Mapping of the Major Histocompatibility Complex Region Linked to Leprosy in Northern China

**DOI:** 10.3389/fgene.2021.768259

**Published:** 2021-12-16

**Authors:** Ruixue Zhang, Lu Cao, Weiwei Chen, Huiyao Ge, Xia Hu, Zhuo Li, Yirui Wang, Wencheng Fan, Liang Yong, Yafen Yu, Yiwen Mao, Qi Zhen, Hong Liu, Furen Zhang, Liangdan Sun

**Affiliations:** ^1^ Department of Dermatology, No. 1 Hospital, Anhui Medical University, Hefei, China; ^2^ Institute of Dermatology, Anhui Medical University, Hefei, China; ^3^ Key Laboratory of Dermatology, Anhui Medical University, Ministry of Education, Hefei, China; ^4^ Anhui Provincial Institute of Translational Medicine, Hefei, China; ^5^ Inflammation and Immune Mediated Diseases Laboratory of Anhui Province, Hefei, China; ^6^ Shandong Provincial Hospital for Skin Diseases and Shandong Provincial Institute of Dermatology and Venereology, Shandong First Medical University and Shandong Academy of Medical Sciences, Jinan, China

**Keywords:** leprosy, MHC, imputation, single nucleotide polymorphism, copy number variant

## Abstract

**Background:** Leprosy is a chronic infectious skin and neurological disease, and genetic background is considered to be one of the major factors of risk. The major histocompatibility complex (MHC) region not only affects susceptibility to leprosy but also its development and outcome. Given the complex traits of the MHC region, variants and the potential mechanism by which HLA influences leprosy development need to be further explored.

**Methods:** We extracted previous genome-wide association study data from the Northern Han Chinese population to perform HLA fine-mapping. Using the 1,000 Genome Project Phase 3 dataset as the reference panel, single-nucleotide polymorphisms (SNP), insertion and deletion (INDEL) and copy number variant (CNV) imputation were carried out. HLA classical alleles and amino acids in the MHC region were imputed using the HAN-MHC database. Further stepwise regression analysis was conducted to analyze independent signals of variants related to leprosy.

**Results:** We identified four independent variants: esv3608598, rs7754498, rs3130781 and rs144388449. Among them, esv3608598 is a CNV and the first HLA CNV associated with leprosy risk. SNP annotation using RegulomeDB, HaploReg, and rVarBase showed that three SNPs are likely to affect the pathogenesis of leprosy.

**Conclusion:** In summary, this is the first study to assess the association between HLA CNV and leprosy susceptibility in a Northern Han Chinese population. By fine mapping of the MHC region in this population, our findings provide evidence for the contribution of HLA to leprosy susceptibility.

## Introduction

Leprosy, or Hansen’s disease, is a chronic infectious skin and neurological disease caused by *Mycobacterium leprae*. In addition to the skin and peripheral nervous system, it can affect the eyes, bones, mucous membranes, testes and other body parts to a certain extent (White and Franco-Paredes, 2015). Although more than six million people have been cured by multidrug therapy since the 1980s, leprosy is still an important public health problem in many regions of the world (Moraes and Düppre, 2021). Indeed, the World Health Organization (WHO) reported 202,185 new cases of leprosy in 2019 (WHO, 2020).

Ridley and Jopling proposed in 1966 that leprosy can be divided into five main clinical forms according to the immune response, histopathology and bacterial factors (4). Tuberculoid leprosy, at one end of the clinical spectrum, involves an immune response mediated by Th1 cells, which is characterized by limited skin lesions and a small number of bacteria. At the other end is lepromatous leprosy, with weak immunity, that is characterized by a large number of lesions and intense growth of the *bacillus*. Between the two ends of the spectrum are borderline tuberculoid leprosy, mid-borderline leprosy and borderline lepromatous leprosy, immune-instability forms. In the early stage, indeterminate leprosy can evolve into any of the above forms or heal spontaneously (Ridley and Jopling, 1966). To standardize clinical treatment, the WHO divides leprosy into multibacillary and paucibacillary types (WHO, 1994), (Parkash, 2009).

*M. leprae* is an intracellular acid-fast pathogen that cannot grow in culture medium and is therefore strongly dependent on the host cell environment. Host-pathogen interactions in leprosy over thousands of years have caused *M. leprae* to lose part of its genome. However, the bacterium can still invade human macrophages and Schwann cells (Misch et al., 2010). In fact, only a small percentage of people infected will develop leprosy (Moet et al., 2006). Environmental factors, pathogen burden, and human genetic susceptibility may be responsible for clinical leprosy caused by exposure to *M. leprae*. The extremely low rate of leprosy suggests that the genetic background of the host is an important aspect of susceptibility (Cole et al., 2001).

With the development and popularization of molecular biotechnology, genome-wide association studies (GWASs) and further fine-mapping analysis in specific regions have become important methods to study leprosy susceptibility genes (Fava et al., 2020)*.* Studies in different populations, such as Brazil and India, have found that the major histocompatibility complex (MHC) region is associated with the risk of leprosy (Covolo de Souza-Santana et al., 2021), (Alter et al., 2011). MHC, also known as human leukocyte antigen (HLA), is the most polymorphic genomic region and correlated strongly with the immune system. HLA-DRB1 is the most closely related gene to leprosy per se as well as clinical forms of the disease. HLA-A, HLA-B, HLA-DQB1 and HLA-DQA1 alleles have also been reported to be associated with leprosy (Fava et al., 2020). In China, a genome-wide association study of leprosy showed that HLA-DRB1*15 was the most significant risk allele (Wang et al., 2016). Nevertheless, due to MHC complex genetic characteristics, elucidating the exact role of MHC in leprosy is challenging.

Copy number variant (CNV) is a type of structural variant that affects DNA copy number, such as large deletions and duplications. CNVs affect approximately 12% of the human genome (Redon et al., 2006). Overall, CNVs are associated with a variety of diseases via various molecular mechanisms, such as Mendelian diseases, HIV susceptibility and autoimmunity (Zhang et al., 2009a). To our knowledge, the association between HLA CNV and leprosy susceptibility remains unclear. To identify more MHC loci, including CNVs, SNPs, HLA alleles and amino acid polymorphisms, associated with leprosy in the Chinese Northern Han population, we extracted MHC data from a previous GWAS including 1,363 leprosy patients and 1,238 controls and conducted HLA imputation based on a large Han-MHC reference panel.

## Methods

### Study Participants

We used northern population data from a previous genome-wide association study (Wang et al., 2016), (Liu et al., 2015). In short, leprosy samples were collected from dermatology clinics of various hospitals, and all cases were diagnosed by at least two experts. The controls were selected as individuals who did not have histories of leprosy, autoimmune, or systemic disorders or a family history of leprosy (among first-, second-, or third-degree relatives). Patients and controls were matched according to ethnic origin and geographic region of recruitment. The study subjects were homogeneous, and there was no systematic bias or potential population stratification. The study was approved by the ethics committee of the local institutions and conducted in accordance with the Declaration of Helsinki. Informed consent was obtained from all subjects or their family members. All participants were Northern Han Chinese.

### HLA Imputation and Quality Control

We extracted the SNP genotypes located in the MHC region (from 24 to 36 Mb on chromosome 6) from the GWAS data. Based on two reference panels: the 1,000 Genomes Project Phase 3 reference data and the HLA reference panel of the Chinese Han population (10,689 individuals) (Zhou et al., 2016) constructed in the previous study, HLA imputation was performed using the software Beagle 4.1. (Browning and Browning, 2007). To decrease the real computing time and ensure the imputation process was successful, we split each chromosome into chunks and imputed them in parallel in multiple computer processors. For post-imputed quality control, we obtained high-quality variants which satisfied: 1) call rate of >95%; 2) MAF >0.01; 3) Hardy-Weinberg test *p* value of >1.0 × 10^−3^; 4) imputation dosage Rsquared value of >0.5 for CNVs, imputation dosage R-squared value > 0.9 for SNPs, and imputation dosage R-squared value >0.7 for HLA amino acids and alleles.

### Association and Stepwise Regression Analyses of the MHC Region

For the association study, the biallelic variants were encoded as allele 1 and allele 2, and the multiallelic variants including multi-residue positions and HLA alleles were encoded as the presence or absence of an individual allele. Stepwise regression analysis was conducted to analyze independent signals of variants related to leprosy. In each conditional regression, the top variant was used as a covariate in the model until there were no significant variants. When the most significant variant was an SNP, HLA alleles, amino acids and CNVs with strong LD associated with it were preferred for stepwise regression analysis; otherwise, we selected the SNP as a covariate. All analyses were performed with PLINK 1.9 software (https://www.cog-genomics.org/plink2).

### In Silico Bioinformatics Analysis

To investigate the function of SNPs, we performed bioinformatics analysis using three online prediction websites: HaploReg (http://pubs.broadinstitute.org/mammals/haploreg/haploreg.php), RegulomeDB (http://regulomedb.org/) and rVarBase (http://rv.psych.ac.cn/). HaploReg was employed to explore noncoding genomic annotations for variants and to identify their potential causal relationship with the pathogenesis of the disease (Ward and Kellis, 2016). RegulomeDB was used to annotate variants with regulatory elements by providing scores (Boyle et al., 2012). The rVarBase database annotates SNPs from three aspects: chromatin states, overlapping regulatory elements and potential target genes (Guo et al., 2016).

## Results

### HLA Imputation and Association Analyses

After applying quality control, a total of 1,363 leprosy cases and 1,238 controls were included in this study. There were 36,006 high-quality variants after postimputation quality control. Based on Bonferroni adjustment, *p* < 1.39 × 10^−6^ (0.05/36,006) was considered the study-wide significance threshold. The top three significant variants were rs9271147, esv3608598 (*p* = 2.18 × 10^−25^; OR = 1.98; 95% CI, 1.74–2.25) and DRB1*1501 (*p* = 4.12 × 10^−27^; OR = 2.30; 95%CI, 1.98–2.68). After condition analysis, esv3608598 showed strong LD with rs9271471 and DRB1*1501.

### Stepwise Logistic Regression

**FIGURE 1:**
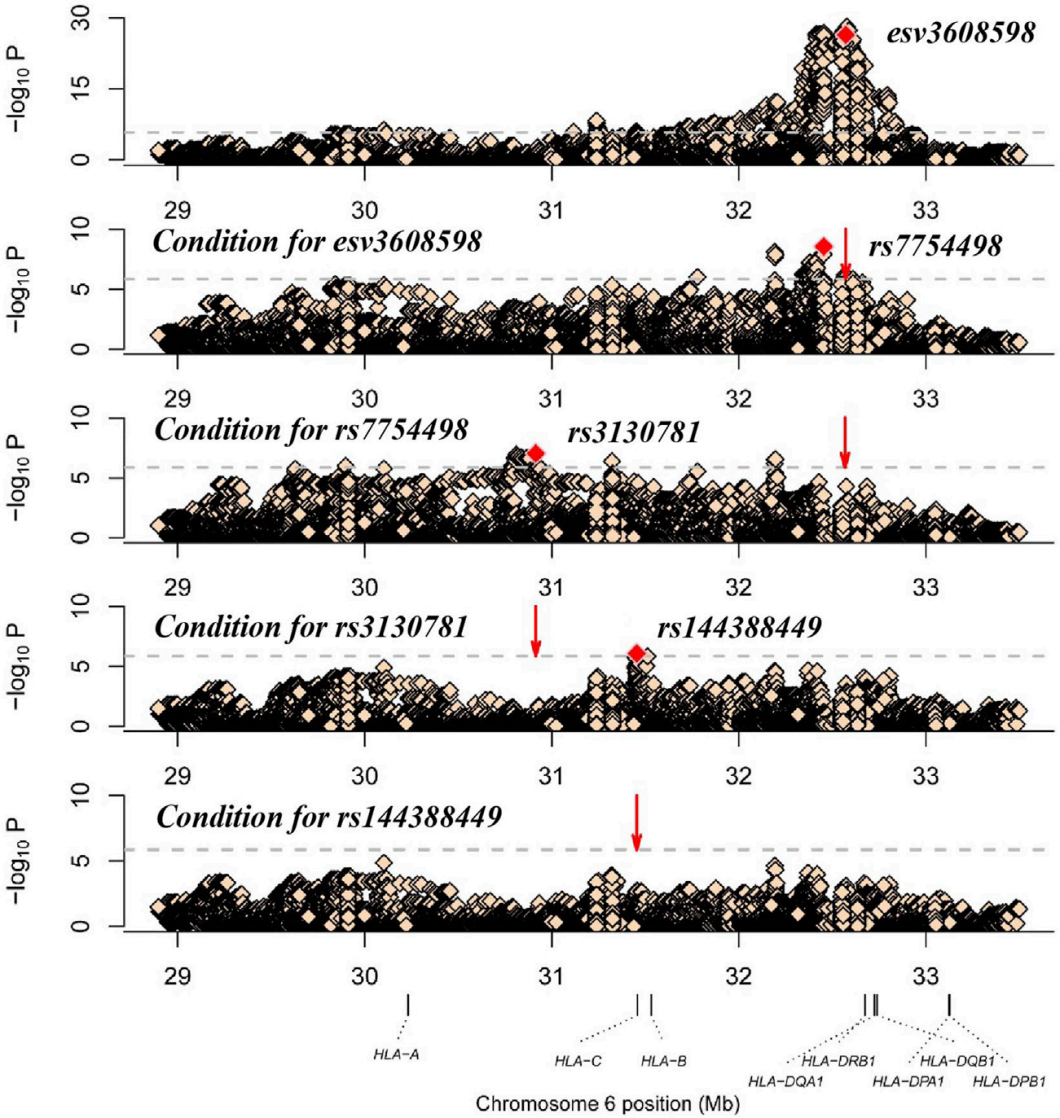
Analysis of the HLA associated with leprosy. The *x*-axis shows genomic position and the *y*-axis shows–log10(*p* value) of the variants. The dashed horizontal line represents the significance threshold of *p* = 1.39 × 10^−6^. The dots marked red in each panel represent the sites used for condition analysis (esv3608598, rs7754498, rs3130781 and rs144388449).

To identify independent signals with variants in the MHC region, we chose the strongest associated CNV (esv3608598) to conduct stepwise logistic regression. Conditioning on esv3608598 revealed a strong second association with rs7754498 (*p* = 2.75 × 10^−9^, OR = 1.42, 95%CI 1.27–1.60). After further condition analysis of rs7754498, the most significant locus was rs3130781 (*p* = 9.25 × 10^−8^, or = 1.6, 95% CI 1.34–1.90). When rs3130781 was added to the covariate for logistic regression analysis, rs1443884 was most significant (*p* = 8.25 × 10^−7^, OR = 0.69, 95%CI 0.60–0.80) (Figure 1). However, no variant satisfied the significance threshold after conditioning of all the above loci. Thus, esv3608598, rs7754498, rs3130781 and rs144388449 may influence leprosy susceptibility independently (Table1).

**TABLE 1:** Stepwise logistic regression of variants associated with leprosy.

STEP	Variant	Location (hg19)	Variant type	Raw				Stepwise analysis after adjusting		Gene annotation	
				Case	Control	OR (95% CI)	*p* Value	OR (95% CI)	*p* Value		
1	esv3608598	Chr6:32571334_32571609	CNV	0.35	0.22	1.98 (1.74–2.25)	2.18 × 10^−25^	NA	NA		Intergenic*HLA-DRB1*, *HLA-DQA1*
2	rs7754498	Chr6:32453500	SNP	0.55	0.43	1.62 (1.45–1.81)	1.68 × 10^−17^	1.42 (1.27–1.60)	2.75 × 10^−9^		Intergenic*HLA-DRA*, *HLA-DRB5*
3	rs3130781	Chr6:30914552	SNP	0.14	0.12	1.15 (0.98–1.35)	0.084	1.6 (1.34–1.90)	9.25 × 10^−8^		Intronic *DPCR1*
4	rs144388449	Chr6: 31454104	SNP	0.23	0.29	0.74 (0.65–0.84)	1.68 × 10^−6^	0.69 (0.60–0.80)	8.25 × 10^−7^		Intergenic*HCG26*, *MICB*

### Functional Annotation

The results of biological functional annotation are shown in Table 2. The SNP rs7754498 is located 32 kb downstream of *HLA-DRB5*. The Regulome DB score shows that rs7754498 may be associated with transcription factor binding or DNase peak. The SNP rs3130781 is located in the intron region of *DPCR1* and rs144388449 is located 8 kb upstream of *MICB*. The scores for rs3130781 and rs144388449 provided by Regulome DB were 1f and 2b, respectively, indicating that the two SNPs may be involved in transcription factor binding and DNase peaks. According to HaploReg, three SNPs are related to the regulation of enhancer, promoter and motif changes. Moreover, according to rVarBase results, *HCG21*, *GTF2H4* and *VARS2* are target genes of rs3130781.

**TABLE 2:** Functional annotation from bioinformatics analysis.

SNP	Regulome DB	HaploReg v3.0				rVarBase		
	Score	Enhancer histone marks	Proteins bound	Motifs changed	GENCODEgenes	Chromatin state^a^	TF binding	rSNPs/rCNVs
rs7754498	5	GI	—	—	32 kb 3′ of *HLA-DRB5*	1	0	329
rs3130781	1f	GI	—	Ets	*DPCR1* intronic	4	0	21
rs144388449	2b	—	CTCF	AP-1,ERalpha-a,SREBP	8.6 kb 5′ of *MICB*	33	0	147

Regulome DB, score: 5:TF, binding or DNase, peak; 1f: eQTL + TF, binding/DNase, peak; 2b:TF, binding + any motif + DNase, Footprint + DNase, peak.

SNP: Single Nucleotide Polymorphism.

a Evidence about the chromatin state of the surrounding region.

## Discussion

Previous GWASs have reported more than 30 independent variants and genes related to leprosy, confirming that the host genetic background plays an important role in leprosy susceptibility. Most leprosy-related genes are associated with immunity, which is consistent with the fact that leprosy is caused by pathogen infection. The MHC region involved in immune responses has also been reported to be associated with leprosy in different populations (Wong et al., 2010; Jarduli et al., 2013). Fine mapping studies of the MHC region have been successfully applied to many diseases, such as systemic lupus erythematosus, psoriasis and vitiligo (Okada et al., 2014; Hanscombe et al., 2018; Yang et al., 2018). On the basis of previous GWAS data, we used Beagle 4.1, an HLA-specific imputation tool, the 1,000 Genome Project Phase 3 dataset and the Han-MHC dataset to conduct HLA imputation and identified four independent variants that may explain the associated of leprosy susceptibility with the MHC region.

In the first leprosy GWAS in Han Chinese individuals, the highest risk was conferred by an SNP at the *HLA-DR-DQ* locus: rs602875 (Zhang et al., 2009b). Our team previously used the Han-MHC database as a reference panel for fine mapping and identified four independent sites associated with leprosy (*HLA-DRβ1* amino acid Tyr26, HLA-C*08:01, HLA-DQA1*03:03) (Zhang et al., 2019). In this study, we observed that a CNV associated with leprosy can further reveal links between the MHC region and leprosy. The CNV esv3608598 is a deletion in the intergenic region of *HLA-DRB1* and *HLA-DQA1*, both of which have been reported to be associated with leprosy risk in many countries (Joko et al., 2000; Vanderborght et al., 2007; Zhang et al., 2009c). In addition, rs9271147 was the strongest variant and showed strong LD with esv3608598.

The SNP rs7754498 is located in the intergenic region of *HLA-DRA* and *HLA-DRB5*. A study of leprosy in Indian population showed that *BTNL2-DRA* intergenic SNPs confer risk for leprosy (Ali et al., 2013). *HLA-DRB5* is reported to be associated with susceptibility to leprosy (Koçak et al., 2002). SNP rs3130781 is located in the intron region of *DPCR1*, which was first identified in 2002 as an MHC class I molecule. *DPCR1* modulates NF-κB signaling and plays a role in cell growth (Yan et al., 2018). Moreover, functional annotations by three websites showed that rs3130781 may change DNA motifs and have regulatory effects on transcription. In northern China, *DPCR1* rs2844695 confers a high risk of esophageal squamous cell carcinoma (Shen et al., 2014). However, the association between *DPCR1* variations and risk of leprosy has not been reported. We think rs3130781 may be a significant variant in future studies to validate the function of *DPCR1*. The SNP (rs144388449) is located in the intergenic region of *HCG26* and *MICB*. The latter gene, also known as MHC class I polypeptide-related sequence B, encodes a heavily glycosylated protein involved in both innate and adaptive immunity and is located in the MHC class I region. Previous studies have indicated that *MICB* is closely related to the occurrence of tuberculous leprosy (Tosh et al., 2006). Therefore, our findings suggest that the above three SNPs may be involved in the immune function of MHC and participate in the pathogenesis of leprosy.

There are some limitations in our study. First, the contribution of rare variants (MAF < 0.01) to leprosy was not considered due to the small sample size. Second, HLA variants were not tested in a replication set. In addition, further studies are needed to uncover the underlying mechanism of the MHC region and to confirm its functional role in leprosy.

In conclusion, this is the first study to assess the association between HLA CNV and leprosy susceptibility in a Chinese Northern Han population. In addition to a new CNV, our study reports three independent SNPs in the MHC region that are associated with leprosy risk. The variants we identified emphasize the relationship between host genetic factors and leprosy.

## Data Availability

The original contributions presented in the study are included in the article, further inquiries can be directed to the corresponding author
